# Pediatric scrotal lymphatic malformation: a 15-case series and a proposed diagnostic and management algorithm

**DOI:** 10.3389/fped.2026.1898362

**Published:** 2026-09-09

**Authors:** Lei Kang, Guangyu Zhu, Rui Jia, Tao Guo, Gaofeng Zhang, Ming Bai

**Affiliations:** Department of Urology, Xi’an Children’s Hospital, Xi’an, China

**Keywords:** scrotal lymphatic malformation, pediatric, misdiagnosis, diagnostic algorithm, surgical management, sclerotherapy

## Abstract

**Objective:**

To investigate the clinical characteristics, risks of misdiagnosis, and diagnostic and management strategies for pediatric scrotal lymphatic malformation.

**Methods:**

A retrospective analysis was conducted on clinical data (demographics, symptoms, diagnostic work-up, and treatment) of 15 pediatric cases with scrotal lymphatic malformation treated at our institution between 2012 and 2025. Additionally, a targeted literature review of previously reported cases with diagnostic challenges was performed.

**Results:**

All lesions showed a characteristic extratesticular distribution outside the tunica vaginalis. The overall accuracy rates of preoperative color Doppler ultrasound and clinical diagnosis were only 46.7% (7/15) and 40.0% (6/15), respectively. Furthermore, 26.7% (4/15) of cases had an indeterminate preoperative diagnosis, and 33.3% (5/15) were completely misdiagnosed. Surgical excision was performed in all patients, achieving complete resection in 10 cases and incomplete resection in 5 cases. Over a follow-up period ranging from 3 months to 6 years (mean, 22 months), 3 cases developed ipsilateral scrotal or inguinal recurrence, and 1 case presented with a gluteal lymphatic malformation one year postoperatively. A literature review identified an additional 12 reported cases with incorrect or indeterminate preoperative diagnoses. Indeterminate diagnosis was the largest category (41.7%), and among the definite misdiagnoses, hydrocele was the most common (25%), which closely mirrored the pattern observed in our series.

**Conclusion:**

Pediatric scrotal lymphatic malformation is a rare entity that is highly prone to misdiagnosis and recurrence. Based on our 15-case series and reported cases, we propose a diagnostic and management algorithm integrating clinical evaluation, stepwise imaging, surgical excision, and selective sclerotherapy to improve diagnostic accuracy and optimize surgical outcomes.

## 1 Introduction

Lymphatic malformations (LMs) are benign lesions that result from abnormal development of the lymphatic system, typically occurring in lymphatic-rich areas such as the head, neck, and axilla. Historically, these lesions were often referred to as “lymphangiomas”, but the term “lymphatic malformation” is now preferred according to the latest International Society for the Study of Vascular Anomalies (ISSVA) classification. Scrotal lymphatic malformation (SLM), however, is exceptionally rare, with only sporadic pediatric cases reported in the literature (1–4). Due to its atypical location and clinical features that mimic other pediatric scrotal diseases, childhood SLM is highly prone to misdiagnosis; some patients even undergo multiple inappropriate surgeries before an accurate diagnosis is established (5). This study analyzed the data of pediatric patients with SLM treated at our hospital over a 13-year period. By combining with relevant literature, it explored the clinical characteristics, misdiagnosis risks, and diagnostic and therapeutic strategies for pediatric SLM, ultimately proposing a diagnostic and management algorithm.

## 2 Materials and methods

### 2.1 Study design

This was a single-center, retrospective descriptive observational cohort study of 15 male patients with pathologically confirmed SLM who were admitted to our hospital between February 2012 and February 2025. The study was conducted in accordance with the Declaration of Helsinki and approved by the review board of Xi’an Children’s Hospital (approval number: 20250008). Informed consent for this retrospective analysis was obtained from the guardians of the children.

### 2.2 Data collection

Demographic, clinical, imaging and pathological data were retrospectively collected from the hospital’s electronic medical records. Lesion sizes were uniformly measured in 3 dimensions (L × W × H) based on preoperative ultrasound for all cases. Two independent researchers extracted the relevant data, including age, laterality, clinical presentation, disease duration, preoperative imaging findings [color Doppler ultrasound, computed tomography (CT), magnetic resonance imaging (MRI)] and the resulting preoperative clinical diagnoses, intraoperative findings, pathological results, intraoperative and postoperative management, and follow-up outcomes. All collected data were cross-checked for accuracy and entered into a structured database (Microsoft Excel).

### 2.3 Diagnostic evaluation

All patients underwent preoperative scrotal and inguinal color Doppler ultrasound. CT and/or MRI was performed in selected cases at the discretion of the attending surgeon. Serum alpha-fetoprotein (AFP) was tested in cases with indeterminate lesion characteristics. Integrating the findings of these auxiliary investigations with detailed history and physical examination, the attending surgeon established the preoperative clinical diagnoses.

### 2.4 Treatment and follow-up

All patients in this series underwent surgical intervention due to symptomatic presentation, progressive lesion enlargement, or diagnostic uncertainty requiring pathological confirmation. For patients with a preoperative diagnosis of SLM, a scrotal or combined inguinal-scrotal approach was selected according to lesion location and extent. For patients with incorrect or indeterminate preoperative diagnoses, the surgical strategy was revised intraoperatively upon identification of characteristic cystic lymphatic tissue, and LM excision was subsequently performed. Complete macroscopic resection was attempted in all cases while preserving the testis, spermatic cord, and adjacent vital structures. In cases where complete resection was not feasible, intraoperative sclerotherapy with pingyangmycin (bleomycin A5) was administered to the residual lesions. The protocol involved a concentration of 1 mg/mL pingyangmycin, which was injected directly into any remaining cystic cavities or, if no complete cyst was present, into the base of the residual lesions. The total dosage did not exceed 0.5 mg/kg body weight. Postoperative follow-up included clinical examination and color Doppler ultrasound at regular intervals.

### 2.5 Literature search strategy

To contextualize our findings and identify relevant comparative cases, a focused literature search of the PubMed and Embase databases was conducted. The search aimed to identify previously reported cases of pediatric SLM with incorrect or indeterminate preoperative diagnoses published from 2000 to 2025. The specific search strategy employed was: (“scrotal lymphatic malformation” OR “scrotal lymphangioma”) AND (“pediatrics” OR “child”). No language restrictions were applied. Inclusion criteria comprised reports of primary scrotal lymphatic malformation. Exclusion criteria included reports with accurate preoperative diagnoses and duplicate reports. Two independent researchers (Kang L and Zhu G) screened the titles and abstracts for relevance, and full texts of potentially eligible articles were retrieved and reviewed to extract pertinent clinical, diagnostic, and outcome data.

### 2.6 Statistical analysis

Descriptive statistics were used to summarize the patient characteristics and outcomes. Continuous variables were presented as mean ± standard deviation (SD) as appropriate, while categorical variables were presented as frequencies and percentages. All statistical analyses were performed using Microsoft Excel.

## 3 Results

### 3.1 Patient demographics and clinical presentation

A total of 15 male patients were included. The mean age at presentation was 5.8 ± 2.8 years (range: 0–12 years). The lesion was located on the left side in 7 cases and on the right side in 8 cases. The predominant clinical presentation was a painless scrotal mass (11/15, 73.3%). However, 4 patients (26.7%) presented with scrotal erythema, swelling, and pain. Notably, all 4 of these acute cases had a recent history of scrotal trauma. Disease duration varied widely, from 1 day to over 5 years. In acute symptomatic cases, disease duration was calculated from symptom onset, as prior history could not be reliably ascertained. One patient had a history of recurrence following a prior resection at another institution.

### 3.2 Preoperative imaging and clinical diagnoses

Preoperative color Doppler ultrasound correctly suggested SLM in only 7 of the 15 cases (46.7%). Among the remaining 8 cases, 4 were reported as indeterminate scrotal masses with indeterminate features, and 4 were incorrectly diagnosed as hydrocele (*n* = 2), scrotal hematoma (*n* = 1), or scrotal hematoma with testicular necrosis (*n* = 1). Notably, the misdiagnoses of hydrocele occurred in cases where the predominantly anechoic cystic nature masked the presence of internal complex architecture or subtle septations. Of the 4 patients who underwent CT, the findings concurred with ultrasound in 3 cases but reclassified one ultrasound-suggested LM as a hydrocele, highlighting the variability in imaging interpretation. In the 2 patients who underwent MRI, both diagnoses were consistent with ultrasound, indicating LM and providing better anatomical delineation of lesion extent.

Based on all available preoperative information, a correct clinical diagnosis of SLM was made in only 6 of 15 cases (40.0%). Among the remaining 9 patients, 5 were definitively misdiagnosed—as simple hydrocele (*n* = 2), acute scrotum (*n* = 2), or scrotal hematoma (*n* = 1)—while 4 had indeterminate diagnoses that could not be further characterized preoperatively. Serum AFP testing in the 4 indeterminate cases was unremarkable. Detailed preoperative diagnostic data for all 15 patients are presented (Table 1).

**Table 1:** Demographic, clinical, imaging, and pathological data of this group of cases.

No.	Age (Years)	Symptom duration (Months)	Clinical presentation	Side	Mass size (cm)	Relevant trauma history	Ultrasound Diagnosis	CT Diagnosis	MRI Diagnosis	AFP	Preoperative clinical diagnosis	Pathological Diagnosis
1	8.3	60	Painless scrotal and inguinal mass	Right	4 × 3 × 3	No	LM	/	/	/	LM	Cystic LM
2	5.1	1	Painless scrotal and inguinal mass	Left	5 × 3 × 2	No	LM	LM	/	/	LM	Cystic LM
3^a^	7	3	Painless scrotal mass	Left	1.5 × 1 × 1	No	LM	/	/	/	LM	Cystic LM
4	7.2	1	Painful scrotal mass	Right	3 × 2 × 2	Yes	LM with hemorrhage	/	LM with hemorrhage	/	LM with hemorrhage	Cystic LM with hemorrhage
5	6.9	0.25	Painless scrotal mass	Right	5 × 5 × 4	No	LM with hemorrhage	/	LM with hemorrhage	/	LM with hemorrhage	Cystic LM with hemorrhage
6^b^	5.7	0.33	Painless scrotal mass	Left	4 × 3 × 3	No	Hydrocele	/	/	/	LM	Cystic LM
7	1.3	12	Painless scrotal mass	Right	8 × 5 × 4	No	Hydrocele	/	/	/	Hydrocele	Cystic LM
8	4.1	6	Painless scrotal mass	Right	5 × 2 × 2	No	LM	Hydrocele	/	/	Hydrocele	Cystic LM
9	3.9	6	Painless scrotal mass	Right	6 × 4 × 3	No	Indeterminate scrotal mass	/	/	Normal	Indeterminate scrotal mass	Cystic LM
10	5.1	12	Painless scrotal mass	Left	5 × 4 × 3	No	Indeterminate scrotal mass	Indeterminate scrotal mass	/	Normal	Indeterminate scrotal mass	Cystic LM
11	0.4	4	Painless scrotal mass	Right	1 × 1 × 1	No	Indeterminate scrotal mass	/	/	Normal	Indeterminate scrotal mass	Cystic LM
12	12	3	Painless scrotal mass	Left	2 × 1 × 0.4	No	Indeterminate scrotal mass	Indeterminate scrotal mass	/	Normal	Indeterminate scrotal mass	Cystic LM
13	6.3	0.03	Scrotal redness, swelling, and pain	Left	5 × 4 × 3	Yes	LM with hemorrhage	/	/	/	Acute scrotum	Cystic LM
14	5.7	0.1	Scrotal redness, swelling, and pain	Left	6 × 2 × 2	Yes	Hematoma with testicular necrosis	/	/	/	Acute scrotum	Cystic LM with hemorrhage
15	8.3	0.03	Scrotal redness, swelling, and pain	Right	4 × 3 × 3	Yes	Hematoma	/	/	/	Hematoma	Cystic LM with hemorrhage

The symbol “/” indicates that the examination was not performed.

a Case 3 is a recurrent case, having undergone ipsilateral SLM resection 4 years prior.

b Case 6 was initially diagnosed with a hydrocele, and the diagnosis was revised to LM following multiple preoperative physical examinations.

### 3.3 Intraoperative findings and treatment

The surgical approach was frequently influenced by the preoperative diagnosis, often necessitating an intraoperative shift in strategy. Among the 9 patients with an incorrect or indeterminate preoperative diagnosis, the 2 patients preoperatively diagnosed with hydrocele initially underwent surgery for high ligation of the processus vaginalis, which was found to be closed intraoperatively, leading to a revised approach for LM excision. The 2 patients misdiagnosed with acute scrotum, the 1 patient misdiagnosed with hematoma, and the 4 with indeterminate masses all required surgical exploration to establish a definitive intraoperative diagnosis of LM.

Despite the varied preoperative diagnoses, a consistent anatomical pattern was observed across all 15 cases. The lesions were invariably located extratunically, typically enveloping the tunica vaginalis without invading the testis or spermatic cord, which remained morphologically intact. The lesions exhibited multilocular cystic changes and often showed diffuse, infiltrative extension toward the inguinal, prepubic, and perineal regions, with ill-defined lesion margins.

**Figure 1:**
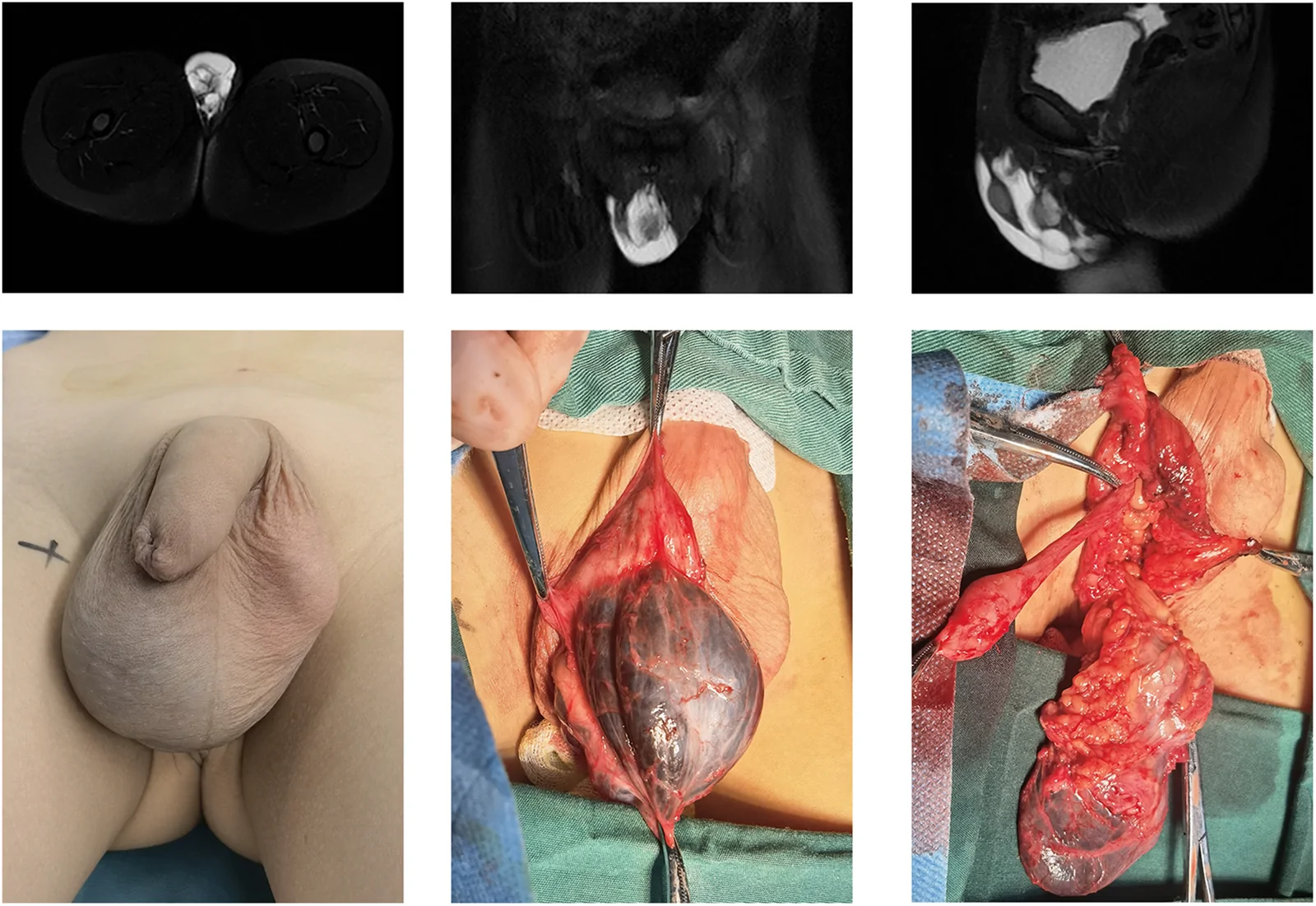
A representative example (case 5): **(A–C)** axial, coronal, and sagittal T2-weighted MRI images demonstrate a multiloculated cystic lesion of the scrotum with internal septations; **(D)** preoperative photograph of the scrotum; **(E)** the lesion completely encapsulates the tunica vaginalis and testis; **(F)** the tunica vaginalis is carefully dissected out, revealing diffuse lesion infiltration towards the scrotal septum, bulbar urethra, and pubic tubercle.

Complete macroscopic resection was achieved in 10 patients (66.7%). In the remaining 5 cases (33.3%), resection was incomplete due to extensive infiltration into surrounding structures. For these 5 patients, intraoperative pingyangmycin was immediately administered to manage the residual lesions and mitigate recurrence. A representative example (Case 5) of such infiltrative disease precluding complete resection is shown (Figure 1).

### 3.4 Pathology and follow-up

**Figure 2:**
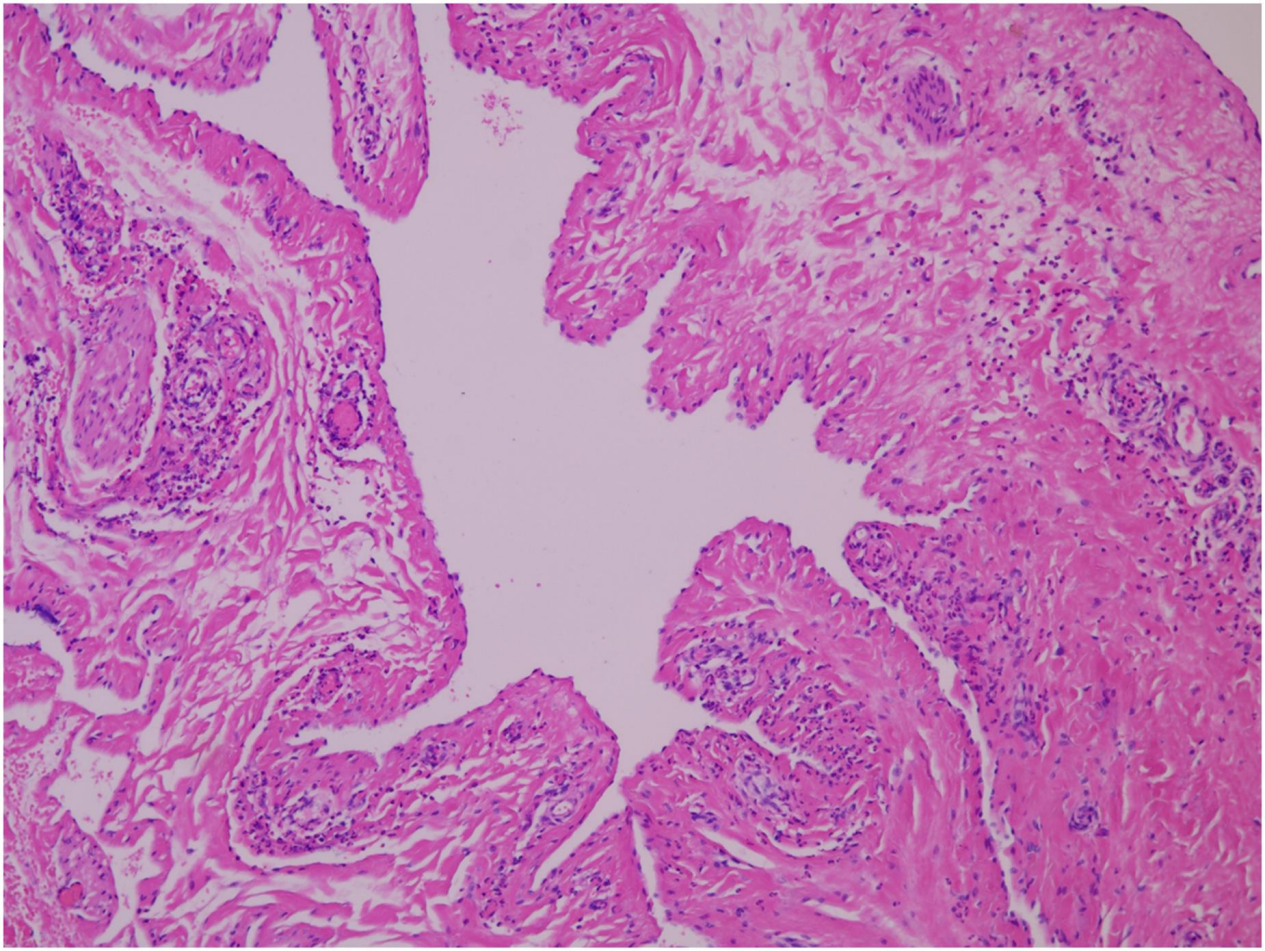
A representative microphotograph of histopathological examination (case 5) (H&E, ×100).

Histopathological examination of resected specimens generally revealed irregularly dilated lymphatic channels lined by flattened endothelial cells, often separated by thin fibrous septa, with varying degrees of perivascular chronic inflammatory cell infiltration (Figure 2).

All 15 patients were followed up until their last recorded clinic visit or contact. The follow-up period ranged from 3 months to 6 years (mean, 22 months). During this period, 4 cases experienced local recurrence or extension of the lymphatic malformation. Two cases presented with ipsilateral scrotal recurrence 1 month postoperatively: one had been considered completely resected during the initial surgery, while the other involved an incomplete resection combined with adjunctive sclerotherapy. A third patient developed an ipsilateral groin recurrence 3 months after an incomplete resection and adjunctive sclerotherapy. These 3 recurrent lesions were small and asymptomatic, managed only with follow-up observation. Additionally, one patient developed an ipsilateral gluteal LM one year postoperatively. This patient’s original scrotal lesion had been deemed completely resected. Given the contiguous nature of the gluteal lesion with the prior scrotal lymphatic malformation, we considered it to be an extension recurrence. This gluteal lymphatic malformation was subsequently surgically resected by the orthopedic department without subsequent sclerotherapy. Of the 5 patients who received adjuvant sclerotherapy following incomplete resection (all as single intraoperative injection without additional sessions), 3 remained recurrence-free throughout their follow-up. No postoperative adverse reactions (such as local necrosis or systemic toxicity) were observed in these patients. Individual treatment and follow-up data are summarized (Table 2).

**Table 2:** Treatment and follow-up data of this group of cases.

No.	Complete macroscopic resection	Adjuvant sclerotherapy	Follow-up (Months)	Recurrence status	Recurrence site	Time to recurrence (Months)	Management of recurrence
1	Yes	No	36	No	/	/	/
2	Yes	No	16	No	/	/	/
3	No	Yes	8	No	/	/	/
4	Yes	No	12	No	/	/	/
5	No	Yes	17	Yes	Ipsilateral scrotum	1 month	observation
6	Yes	No	3	No	/	/	/
7	No	Yes	50	No	/	/	/
8	No	Yes	15	Yes	Ipsilateral groin	3 months	observation
9	Yes	No	18	Yes	Ipsilateral gluteus	12 months	Surgical excision
10	Yes	No	3	No	/	/	/
11	Yes	No	6	No	/	/	/
12	Yes	No	20	No	/	/	/
13	Yes	No	72	No	/	/	/
14	No	Yes	25	No	/	/	/
15	Yes	No	28	Yes	Ipsilateral scrotum	1 month	observation

The symbol “/” indicates not applicable due to no recurrence.

### 3.5 Literature review of incorrect and indeterminate preoperative diagnosed cases

Given the rarity of the disease and the nature of case report publications, which may be subject to publication bias, calculating an absolute overall misdiagnosis rate is neither feasible nor the aim of this review. We focused on characterizing the distribution of incorrect and indeterminate preoperative diagnoses. A total of 12 reported cases with incorrect or indeterminate preoperative diagnoses were identified and summarized (Table 3) (1, 2, 6–15). Analysis of these 12 cases reveals that preoperative evaluations failed to yield a definitive diagnosis in 5 cases, which accounted for the largest proportion (5/12, 41.7%) of the cohort. Among the definitively misdiagnosed cases, hydrocele was the most common erroneous initial diagnosis (3/12, 25%). Furthermore, symptomatic or complicated lesions were frequently misclassified as local emergencies, including acute scrotum (2/12, 16.7%) and scrotal hematoma (1/12, 8.3%). Notably, one case (1/12, 8.3%) was misdiagnosed as an inguinal hernia, undergoing several unsuccessful reduction attempts despite the absence of strangulation signs (8). These literature-derived diagnostic distributions closely mirror the pitfalls observed in our own series, highlighting a frequent diagnostic challenge in the initial assessment of pediatric scrotal masses.

**Table 3:** Reported cases of pediatric SLM with erroneous or non-specific preoperative diagnoses.

NO.	First author (Year)	Age (Year)	Clinical presentation	Trauma history	Preoperative diagnosis	Ultrasound findings	MRI/CT Done	Pathological diagnosis	Treatment
1	Ali AY (2022) (1)	6	Painless, progressive right scrotal mass	No	Hydrocele	Multiple cystic lesions with septa	No	Cystic lymphangioma	Excision via median raphe incision
2	Parveen K (2022) (6)	11	Gradually progressive right scrotal swelling with sudden enlargement and pain	No	Hydrocele	No	No	Lymphangioma	Surgical excision
3	Yap M (2015) (7)	3	Painless, progressive left scrotal mass	No	Hydrocele	Cystic lesion with multiple septa	No	Lymphangioma	Excision via inguinal incision
4	Patoulias I (2014) (8)	3.5	Painless, right scrotal and inguinal mass	No	Inguinal hernia	Cystic lesion encircling spermatic cord	No	Cystic lymphangioma	Excision via inguinal incision
5	Yan S (2025) (2)	13	Perineal pain, tender right scrotal mass	No	Indeterminate scrotal mass	Multiple cystic lesions with septa	Yes (MRI)	Lymphangioma	Surgical excision
6	Kajal P (2013) (9)	1.5	Painless, progressive left scrotal enlargement	No	Indeterminate scrotal mass	Abdominal Ultrasound normal	No	Cystic lymphangioma	Excision via scrotal incision
7	Diakité ML (2011) (10)	2	Cystic mass discovered after scrotal trauma	Yes	Indeterminate scrotal mass	Cystic mass	No	Cystic lymphangioma	Surgical excision
8	Eric R W (2002) (11)	15	Palpable painless left scrotal mass	No	Indeterminate scrotal mass	Cystic lesion with multiple septa	No	Lymphangioma	Surgical excision
9	Mhoon JM (2002) (12)	3.5	Acute right-sided scrotal pain and swelling	Yes	Indeterminate scrotal mass	Cystic areas with multiple septa, hemorrhage	No	Cystic lymphangioma	Excision via inguinal and scrotal incision
10	Ntai S (2011) (13)	13	Acute left-sided scrotal pain and swelling	No	Acute scrotum	Complex septated cyst with mixed internal echogenicity	No	Cystic lymphangioma with hemorrhage	Surgical excision
11	Häcker A (2006) (14)	5	Acute right-sided scrotal pain and swelling	No	Acute scrotum	Septated cystic, non-perfused mass	Yes (MRI)	Lymphangioma	Excision via inguinal and scrotal incision
12	Mercedes N (2002) (15)	13	Right palpable paratesticular mass with scrotal discomfort	Yes	Hematoma	Multi-septated fluid collections in the right scrotum	No	Cystic lymphangioma	Surgical excision

Pathological diagnoses are reproduced as reported in the original publications; the historical term “lymphangioma”corresponds to “lymphatic malformation” in the current ISSVA terminology.

## 4 Discussion

Our series demonstrates the inherent diagnostic vulnerability of pediatric SLM, as both clinical assessment and sonographic evaluation proved to be inadequate as standalone diagnostic modalities, with a substantial proportion of patients experiencing diagnostic uncertainty or complete misdiagnosis preoperatively. Consequently, the principal clinical challenge of pediatric SLM is its high risk of preoperative misdiagnosis, which can lead to inappropriate initial management, incomplete excision, and avoidable recurrences (5, 8, 16).

A central finding of our study is the characteristic extratesticular growth pattern of these lesions. In all 15 patients, the lesion was located outside the tunica vaginalis, mostly enveloping it while leaving the tunica vaginalis and testes morphologically intact (1, 2, 12, 17). This testis-enveloping, fluid-filled nature explains why the lesion is readily mistaken for a simple hydrocele on initial physical examination (6, 18, 19). Meanwhile, its infiltrative and multilocular characteristics, particularly when extending irregularly toward the perineum or inguinal regions, create highly variable and ambiguous sonographic appearances that confound sonographic interpretation and lead to its classification as an indeterminate extratesticular mass (2, 11). The structural bias in evaluating pediatric scrotal disease—where painless masses are assumed to be hydroceles or hernias, and painful lesions are categorized as emergencies like torsion or trauma—contributes to this diagnostic pitfall by narrowing the differential too early (6, 14). Strikingly, in one of our misdiagnosed cases, the attending physician proceeded with hydrocele-oriented surgery despite the preoperative ultrasound correctly suggesting a septated LM. This illustrates a profound anchoring bias, where the initial clinical presumption of a routine hydrocele overrode objective sonographic evidence of SLM, underscoring the necessity of a standardized diagnostic algorithm designed to mitigate such diagnostic inertia.

The duality in the clinical presentation of SLM deserves particular emphasis, as it poses a significant diagnostic challenge by mimicking more common acute conditions. Typically, it manifests as a slowly enlarging, painless inguinoscrotal mass (1, 6). However, secondary factors such as intracystic hemorrhage, inflammation, or trauma can abruptly convert an indolent lesion into a painful, swollen, erythematous acute scrotum (6, 13, 18). In our cohort, all 4 children presenting with scrotal erythema and pain had a recent history of trauma. The acute onset of symptoms led to preoperative misclassification as acute scrotum (*n* = 2) or scrotal hematoma (*n* = 1) in 3 of these patients; only 1 was correctly identified as an LM with secondary hemorrhage. This misclassification may reflect how an acute inflammatory response can mask the underlying chronic pathology and how the urgency of the situation focuses attention on common acute etiologies. Therefore, a history of trauma should not prematurely terminate the differential diagnosis; rather, trauma may serve as the event that unmasks a preexisting, silent lymphatic malformation (2, 20). Detailed history-taking and dynamic physical examinations remain indispensable. A long-standing mass that lacks clear fluctuation with posture or crying, exhibits partial compressibility, and appears separable from the testis should prompt reconsideration of a simple hydrocele diagnosis. Furthermore, from an anatomical and developmental perspective, it is important to distinguish primary SLM from lymphatic malformations of retroperitoneal, omental, or mesenteric origin that secondarily extend into the scrotum via a patent processus vaginalis or along the inguinal canal. These intra-abdominal lesions can exhibit postural size fluctuation and positive transillumination, perfectly mimicking a communicating hydrocele or an inguinal hernia (21, 22). Although our case series exclusively comprises primary SLMs without demonstrable intra-abdominal extension, this specific anatomical trap underscores a critical clinical pearl: when evaluating any pediatric scrotal mass, the sonographic examination should be extended to the inguinal canal and, when indicated, the abdominal cavity to rule out a higher anatomical origin that could significantly alter management.

Ultrasonography remains the primary imaging modality for evaluating scrotal masses, but its limitations must be acknowledged, contributing significantly to the observed low diagnostic accuracy for SLM. The classic sonographic appearance of SLM is a multiloculated or septated extratesticular cystic mass (7, 19). The presence of punctate or linear blood flow signals within the thin septa on color Doppler can help distinguish SLM from hydrocele or hematocele, which typically lack true septal vascularity (2, 23). However, spontaneous hemorrhage or infection can profoundly complicate the sonographic appearance: altered internal echogenicity may produce fluid-fluid levels, internal debris, or even pseudo-solid components resembling other pathologies, leading to misinterpretation (13, 15). In our series, the modest sonographic accuracy likely reflects the interpretive challenges posed by hemorrhagic changes, atypical lesion extent, or morphological overlap with septated hydroceles—features that can be particularly difficult for less experienced sonographers to resolve.

In cases where ultrasound is non-diagnostic or the lesion exhibits an atypical or extensive pattern, MRI serves as a highly valuable supplementary tool (24). MRI provides superior soft-tissue contrast, better delineating the multilocular nature of the cyst, the presence of internal hemorrhage (varying signals on T1 and T2-weighted images), and the precise extent of infiltration into adjacent structures such as the perineum, prepubic region, or pelvic spaces (2, 7). This comprehensive anatomical mapping is essential for clarifying ambiguous ultrasound findings and accurately defining the lesion’s extent. While routine use of CT is generally not recommended due to radiation concerns and limited incremental diagnostic value, targeted MRI is crucial for preoperative planning, especially when determining the feasibility of complete surgical resection or selecting appropriate patients for sclerotherapy. It provides an anatomical roadmap that may help minimize treatment-related morbidity and the risk of recurrence (20, 24). Furthermore, normal serum AFP levels in our indeterminate cases reinforced the benign, non-germ cell nature of the lesions, although imaging remains paramount for characterization.

Surgical excision is currently the gold standard for treating pediatric SLM, aiming for immediate lesion removal and pathological confirmation (17). However, our intraoperative findings demonstrate that complete resection is often impeded by the lesion’s tendency to diffusely infiltrate surrounding tissue planes rather than form a well-encapsulated mass (3). In our series, 5 cases required incomplete resection due to extensive infiltration toward the scrotal septum, inguinal region, bulbar urethra, and pubic tubercle. Incomplete excision is the primary driver of recurrence, which can occur locally or in contiguous anatomical regions (e.g., the gluteal recurrence observed in one of our patients) (7). This multifocal or regionally connected disease pattern underscores that SLM should be viewed as part of a broader regional lymphatic malformation spectrum rather than an isolated scrotal entity. This concept is further supported by reported cases of retroperitoneal or mesenteric lymphatic malformations extending into the scrotum through the inguinal canal, blurring the boundary between primary and secondary scrotal lesions, and reinforcing the need for comprehensive preoperative imaging of the entire inguinoscrotal axis (21, 22).

While recurrence-specific data for SLM are limited, existing reports are largely case-based and often include short or undocumented follow-up. Nevertheless, the literature consistently notes a risk of recurrence, most commonly attributing it to incomplete resection and the multifocal, infiltrative nature of the lesion (8, 9). Our series supports this complex pattern: among our 4 recurrent cases, 2 were associated with incomplete resection with adjuvant sclerotherapy. In contrast, the other 2 recurrences occurred in patients in whom complete excision was suggested intraoperatively, highlighting the limitations of macroscopic assessment for this diffusely infiltrative condition. Furthermore, as studies on lymphatic malformations have reported recurrences occurring even decades post-resection, long-term structured sonographic surveillance is recommended for these patients with SLMs (25).

Given the anatomical constraints and the risks of damaging the testis or spermatic cord during extensive surgical dissection, sclerotherapy has emerged as a promising adjunctive or primary treatment modality. Sclerosing agents, such as bleomycin or OK-432, induce a local inflammatory response and subsequent fibrosis, leading to luminal obliteration and volume reduction (3, 20, 26). In our practice, intraoperative application of pingyangmycin for residual tissues was performed in these 5 incompletely resected cases. We observed no postoperative adverse reactions, and 3 of the 5 patients have remained completely recurrence-free during follow-up. Sclerotherapy is particularly advantageous for extensive, multilocular lesions where complete surgical resection is deemed hazardous; however, multiple sessions may be required, and its application must be meticulously guided to avoid neurovascular or testicular injury (3, 26, 27).

**Figure 3:**
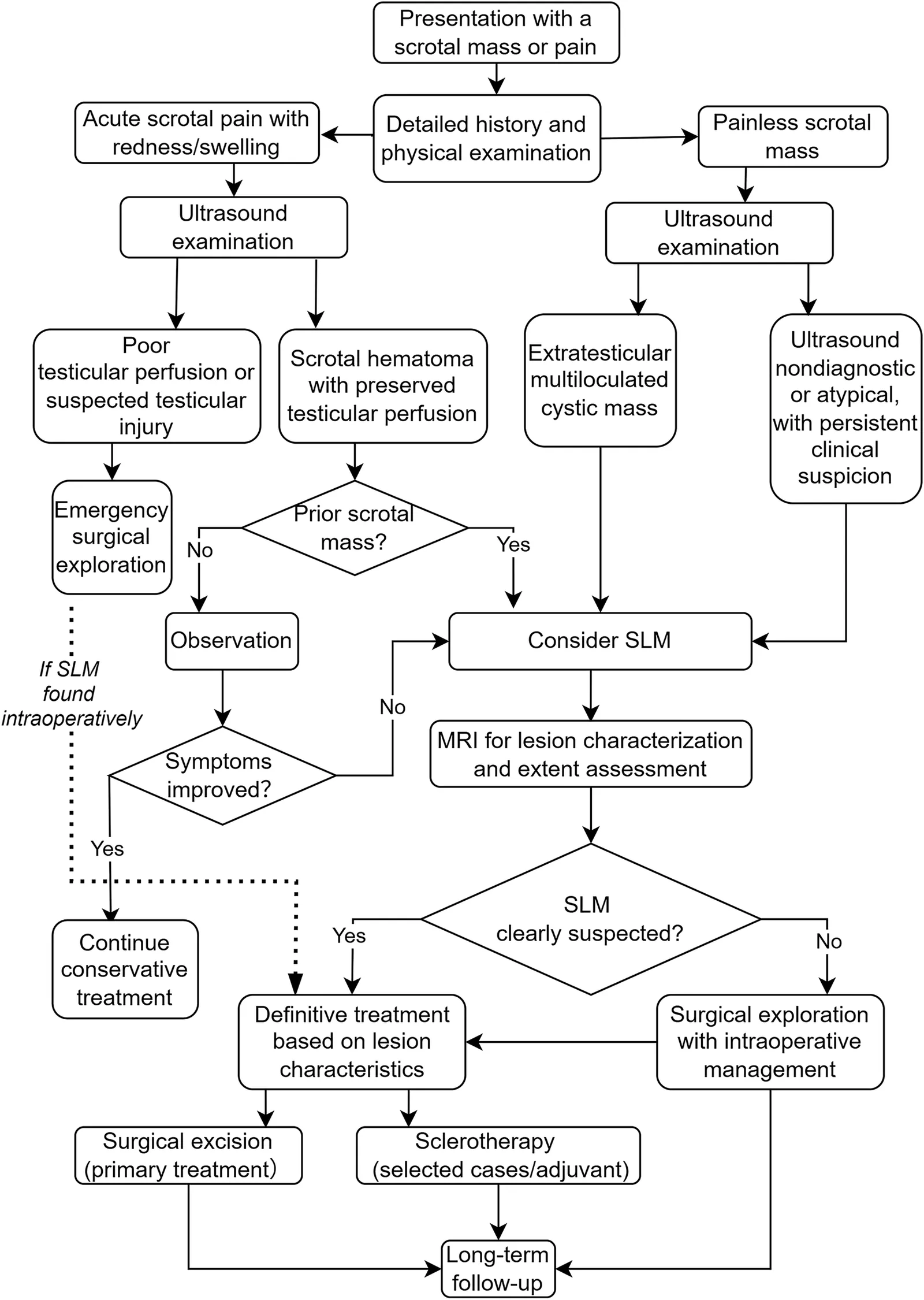
Diagnosis and treatment algorithm for pediatric SLM.

To address the diagnostic and therapeutic challenges of pediatric SLM, we propose a preliminary algorithm (Figure 3). This pathway stratifies patients based on clinical presentation—differentiating between acute, painful presentations requiring immediate exclusion of testicular ischemia, and painless masses where a stepwise imaging approach (ultrasound followed by targeted MRI) is advocated. The algorithm may assist clinicians from initial suspicion through definitive treatment selection, integrating both surgical excision and selective sclerotherapy.

This study has several limitations. First, the retrospective, single-center design and relatively small sample size limit the generalizability of our findings; additionally, formal ISSVA subtyping could not be performed owing to the lack of subtyping information in the original pathology reports. Second, the follow-up period was variable, and longer observation may reveal additional late recurrences. Third, our literature review was confined to case reports and small series with inherent publication and selection biases; calculating a true population-based misdiagnosis rate was therefore not feasible. Finally, the proposed diagnostic algorithm has not yet been prospectively validated.

## 5 Conclusion

Pediatric SLM is a rare condition prone to clinical misdiagnosis and recurrence. Based on our single-center experience and focused literature review, we propose a diagnostic and management algorithm integrating thorough clinical evaluation, pattern-based sonography with selective MRI, structure-preserving surgical excision, and selective sclerotherapy. Long-term follow-up is recommended to monitor for late recurrence. Further prospective, multi-center studies are necessary to validate the proposed algorithm and standardize treatment protocols.

## Data Availability

The original contributions presented in the study are included in the article/Supplementary Material, further inquiries can be directed to the corresponding author.
